# Greater number of weekly stairs climbed is associated with lower low back pain prevalence among female but not male physical therapists

**DOI:** 10.1371/journal.pone.0292489

**Published:** 2023-10-05

**Authors:** Amy H. Amabile, Sharon L. Larson, Lisa T. Hoglund, John P. Guarnieri, Maureen McDonald, Madeline R. Reich

**Affiliations:** 1 Department of Biomedical Education and Data Science, Lewis Katz School of Medicine, Temple University, MERB 457, Philadelphia, PA, United States of America; 2 College of Population Health, Thomas Jefferson University, Philadelphia, Pennsylvania, United States of America; 3 Department of Physical Therapy, Thomas Jefferson University, Philadelphia, Pennsylvania, United States of America; 4 Sidney Kimmel Medical College, Thomas Jefferson University, Philadelphia, Pennsylvania, United States of America; 5 Department of Medical Imaging and Radiation Sciences, Thomas Jefferson University, Philadelphia, Pennsylvania, United States of America; Sohag University Faculty of Medicine, EGYPT

## Abstract

**Introduction:**

Certain cardiovascular health benefits of stair climbing are now widely accepted, but no prior studies have as yet been found linking the quantity of stairs climbed to low back pain (LBP) morbidity. Low back pain is a common musculoskeletal impairment, and research has begun to show an association between LBP and gluteus maximus (GM) weakness. With stair climbing being the activity which most activates GM, the aim of the present research was to assess the relationship between stair ambulation and LBP prevalence. The hypothesis of this cross-sectional study was that individuals with LBP would report a significantly lower numbers of stair flights climbed compared with individuals without LBP.

**Methods:**

A survey tool was developed and distributed via email to a convenience sample of orthopedic physical therapists. Survey items included information regarding medical history, physical activity, workplace, and LBP factors, using a one-year prevalence period.

**Results:**

A total of 363 respondents took the survey and, after application of exclusion criteria, 248 records remained in our final sample. When analyzing all genders together, non LBP (NLBP) respondents reported a mean of 51.62 flights climbed per week; and LBP respondents reported 37.82 flights climbed per week, with *P* = 0.077. When males and females were analyzed separately, a statistically significant difference in mean number of flights of stairs climbed was found among female respondents (61.51 flights climbed for NLBP and 35.61 flights climbed for LBP females; *P* = 0.031). When analyzed based on chronicity of LBP, an even stronger association between stairs climbed and LBP prevalence was found for female respondents with acute LBP (*P* = 0.009).

**Conclusions:**

More weekly stairs climbed was associated with a lower LBP prevalence among females, especially with respect to acute LBP. Randomized, longitudinal research is, however, required to confirm a relationship between stair climbing and LBP.

## Introduction

The potential health impact of stair climbing was first recognized in 1953 with the publication of the London Transport Worker’s Study [1,2], which found a negative correlation between coronary artery disease incidence and multiple types of physical activity, including stairs climbed in the course of a workday. The relationship between stair climbing and cardiovascular morbidity and mortality has continued to be studied over subsequent decades [3–5]; however, no prior studies have as yet been found linking the quantity of stairs climbed to low back pain (LBP) morbidity.

LBP is a common musculoskeletal impairment, with a mean point prevalence among adults worldwide of 8.2% [6], and a 3-month prevalence of 28% among adults in the United States (US) [7]. Total LBP costs, including health care expenses and lost days of productivity, are estimated to range from $46 to $200 billion dollars annually in the US alone [8–10]. In spite of its complex, multifactorial etiology, there is now almost universal agreement among both researchers and clinicians that exercise is one of the most effective treatments for non-specific LBP [11–13]. A wide variety of recommended exercises are seen in the literature [14–16], but many patients do not respond to any intervention, and LBP recurrence rates remain high [17]. There is, therefore, an ongoing need for the development of new, conservative, low-cost, and effective exercise interventions for this problem.

Recent research has begun to show an association between LBP and gluteus maximus (GM) cross-sectional area (CSA) and strength [18–20], but the causal mechanism remains theoretical [21]. Stair climbing is the daily activity which most activates GM [22,23], and if GM size and strength are related to LBP prevalence, the number of stairs climbed should also be associated with LBP prevalence. The aim of the present research was, therefore, to assess the relationship between self-reported stair ambulation and LBP prevalence. Our hypothesis was that individuals with LBP would report a significantly lower number of stair flights climbed compared with individuals without LBP, over a one-year prevalence period.

## Methods

This study was found to be exempt from Institutional Review Board Review (IRB Control #22E.260); however, separate consent forms for both the email survey and focus group, described below, were created and approved by the Thomas Jefferson University Office of Human Research prior to execution of study activities. Our survey was developed using a Qualtrics (Provo, UT) platform, and distributed via email to a convenience sample of physical therapists who were members of the American Physical Therapy Association (APTA) Academy of Orthopedic Physical Therapy (AOPT). Physical therapists were chosen in order to increase the homogeneity in our sample and thereby strengthen the internal validity of our findings. They have a similar socioeconomic and educational level, which have both been shown to be correlated with LBP [24,25]. In addition, physical therapists possess a uniform knowledge base and have a common vocabulary related to patient medical history, LBP definitions, and physical activity-levels, which are all variables of interest in this research. This commonality is derived from a standardized curriculum in US physical therapy schools mandated by the Commission on Accreditation in Physical Therapy Education [26].

The scope of our proposed survey was broad and included multiple topic areas involving medical history, physical activity, workplace factors, as well as LBP prevalence, symptoms, chronicity, and treatment. Realizing that there was no one existing survey tool that met this criteria motivated us to design our own tool. Our team included a trained survey and qualitative researcher (SL) as a co-investigator, and she supervised the design of the tool, and the planning and execution of the focus group of physical therapists who helped to validate it. The survey went through multiple iterations before piloting and validation with our focus group. Although this was an original tool, the inspfiration for our survey items came from multiple sources, including: the National Health Interview Survey [7] for health-related and demographic questions; the Harvard Alumni Study [3] for quantification of stair climbing; the International Physical Activity Questionnaire [27] for questions related to physical activity; McDonald et al’s Physical Activity in Sonographers [28] for questions on work-related activity; the Brief Pain Inventory [29,30] and National Health Interview Survey [7] for pain-related questions; the Knee Injury and Osteoarthritis Outcome Score [31,32] for questions related to functional and recreational activity levels; Skoogh et al’s [33] single item tool for depression and anxiety questions; and Snyder et al’s [34] single item tool for a question on sleep quality.

We chose a one-year prevalence period because it is commonly used in LBP studies [17,35,36], and would allow for us to capture more LBP episodes among our respondents. Definitions of LBP can vary widely and, for the present research, was defined as pain located between the 12th rib and the gluteal fold that may radiate into the proximal or distal lower extremity. This is consistent with Dionne et al’s [37] Delphi Study on LBP definitions, and is a frequently used definition in LBP studies [36,38].

Stratification of LBP cases based on symptom duration varies considerably among researchers. Chronic LBP is almost universally defined as pain lasting more than three months [39]; however, time frames for acute and sub-acute LBP vary considerably [11,38,40–42]. We chose the stratification scheme proposed by Kovacs et al [43], because it was based on a rigorous, multi-center analysis of the appropriate cutoff between acute and subacute pain with respect to patient reported quality of life and disability measures. Final LBP chronicity stratification was as follows: acute pain defined as lasting from 0 through 14 days; subacute pain defined as lasting from 15 days through 12 weeks; and chronic LBP defined as lasting more than 12 weeks.

The survey tool was validated using a focus group of 8 licensed physical therapists working as outpatient therapists within our institution’s health care system. Group participants were recruited by word of mouth through one of the project investigators, and all underwent a prior digital consent, and then a second, verbal consent process at the start of the session. Participants were emailed the link, and took the survey in advance, and the one-hour focus group was conducted via Zoom (Zoom Video Communications, Inc., San Jose CA). Focus group topics included usability, clarity, appropriateness of questions, missing topic areas, and the ability of the tool to reflect possible covariates of LBP. All focus group participants received a $50 gift card to compensate them for participation. Multiple changes were then made to the survey tool based on focus group feedback, including wording changes to increase consistency of response to physical activity and medical history questions, and the addition of items related to psychosocial covariates. A list of the final survey questions is available as an Appendix.

The survey was distributed via a one-time email to the membership list of the APTA AOPT and kept open from 9/7/22 to 10/3/22. Inclusion criteria were: being both a physical therapist and a member of AOPT. Exclusion criteria included: previous spinal, pelvic, or lower extremity fracture; previous major neurological diagnosis; osteoporosis diagnosis; any surgery in the previous 12 months; and > 10 face-to-face or telehealth medical provider visits in last 12 months. This was an anonymous survey, with all data collected being completely deidentified from the respondent. Survey respondents provided consent by clicking on the link in the recruitment email, and were again prompted to consent with the first question in order to continue the survey.

### Statistical analysis

Our power analysis was based on Rey-Lopez’s [3] stair climbing data from the Harvard Alumni Study, which was the best available model from the literature. Sample size calculation was performed using the University of British Columbia online sample size and power calculator [44], with a projected mean between-group difference of 25 flights of stairs climbed per week as a greater effect size; and a difference of 10 flights per week between groups as a lower effect size. This yielded a minimum required size for each sample ranging from 23 respondents to 142 respondents per group, respectively.

Data were analyzed using SPSS version 28.0.1.0 (Armonk, NY) for Windows. A Kolmogorov-Smirnov test for normality was significant for most variables, indicating data were not normally distributed. Our sample size, however, met the accepted threshold which allowed the use of parametric tests for our data analysis [45]. Between group means were compared using a two-tailed Independent samples *t-*test for interval/ratio variables, and applying Levene’s test to assess homogeneity of variance for each measured variable. One-way ANOVA tests with Bonferroni correction were used to compare means when LBP was stratified into 4 groups based on chronicity. Pearson’s Chi-Square test for association was used to compare nominal variables, and Pearson’s product-moment correlation tests were used for all variable correlations. Statistical significance was defined as *P ≤* 0.05.

## Results

The survey was emailed to a total of 15,203 individuals, with 8,941 (58.8%) emails opened, and 363 respondents (4.1%) clicking on and starting to take the survey. Thirty-seven respondents did not complete the survey and their records were removed, leaving a sample size of 326. After application of exclusion criteria, 78 records were removed and 248 records remained in our final sample for analysis.

No significant differences in gender, age, height, weight, body mass index (BMI), years as a physical therapist, and work setting were found between the no LBP (NLBP) and LBP groups (Table 1). A significant difference in full-time versus part-time work status was found between the two groups; however, a Pearson’s product-moment correlation assessing the relationship between hours worked per week and LBP chronicity in our sample showed no correlation (*r*(246) = 0.126; *P* = 0.047), and therefore full-time work status was eliminated from consideration as a covariate.

**Table 1:** Baseline characteristics of participants.

Variable	No Low Back Pain (n = 56)		Low Back Pain (n = 192)		*P*-value
	Mean	Range	Mean	Range	
**Gender Male/Female, n**	26/29	n/a	71/119	n/a	0.186^a^
**Age, years**	43.7	26.0–69.0	42.2	24.0–77.0	0.429^b^
**Height, inches**	66.5	55.0–78.0	66.8	54.0–77.0	0.704^b^
**Weight, pounds**	166.5	75.0–290.0	168.9	102.0–330.0	0.698^b^
**BMI**	26.3	13.7–40.7	26.6	18.1–47.3	0.727^b^
**Years as a Physical Therapist**	17.4	1.0–46.0	16.4	1.0–46.0	0.611^b^
**Work in Outpatient Setting**	46 (82%)	n/a	171 (89%)	n/a	0.168^a^
**Work Full-time** ^**c**^	35 (62%)	n/a	152 (79%)	n/a	0.011^a^

^a^ Pearson Chi-Square

^b^ Independent Samples t-test

^c^ Full-time ≥ 32 hours/week.

Statistical Significance (*P* ≤ 0.05).

### Between group differences in stairs climbed

**Fig 1:**
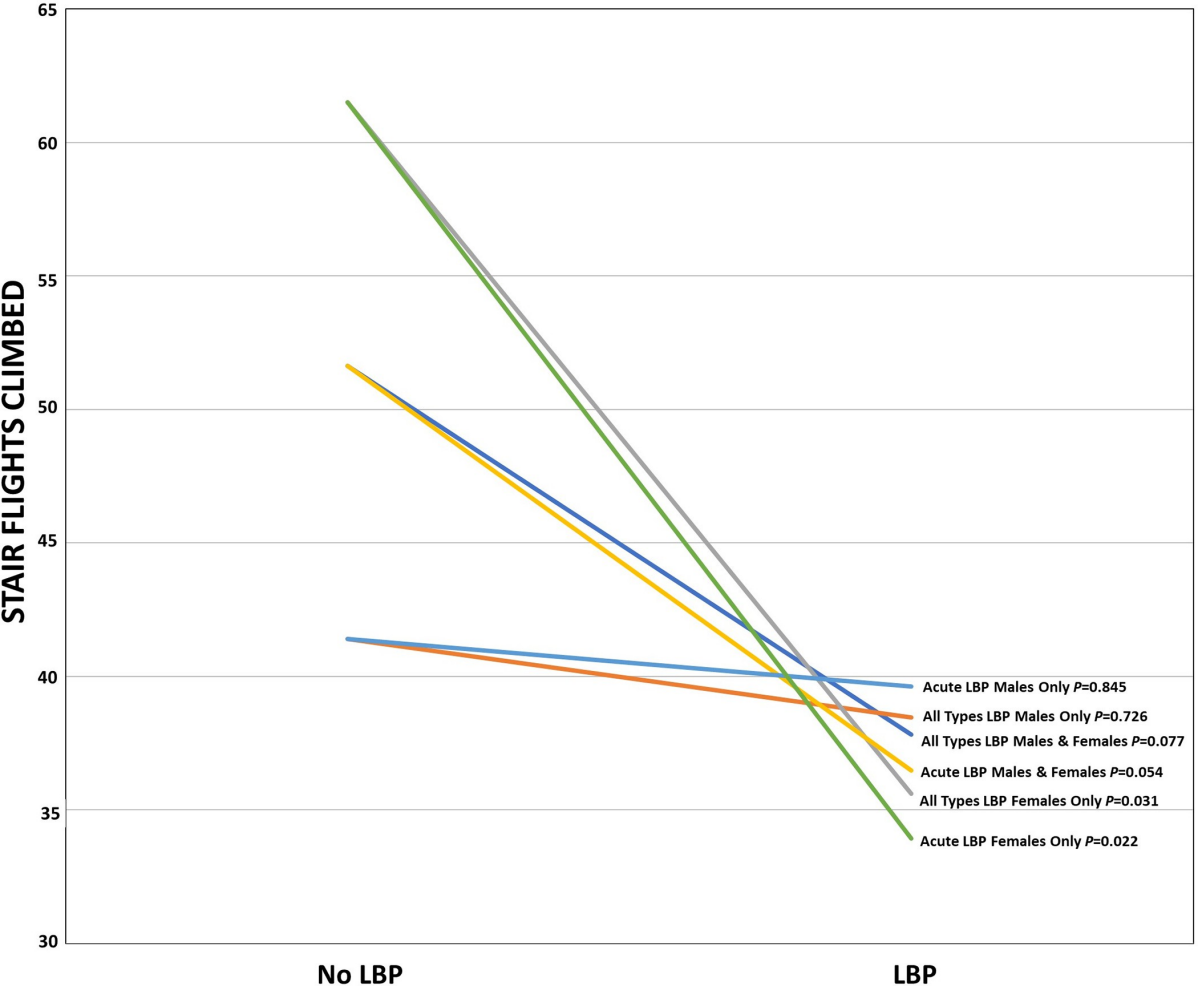
Independent sample t-test results for weekly stair flights climbed for all types LBP and acute LBP by gender.

An independent samples *t*-test was performed to assess differences in mean number of flights of stairs climbed per week between LBP and NLBP respondents. Results of this means testing are summarized in Table 2 and in Fig 1. When analyzing male and female respondents together, NLBP respondents reported a mean of 51.62 (± 54.36; 95% CI, 37.06 to 66.17) flights climbed per week; and LBP respondents reported 37.82 (± 34.53; 95% CI, 32.91 to 42.74) flights climbed per week, (*t*(246) = 1.796; *P* = 0.077).

**Table 2:** Between group differences in weekly stairs climbed.

Variable	No Low Back Pain				Low Back Pain				*P*-value^a^
	n	Mean	Range	95% CI	n	Mean	Range	95% CI	
**Weekly stair flights climbed, males & females (n = 248)**	56	51.62	<10 - >200 flights	37.06–66.17	192	37.82	<10 - >200 flights	32.90–42.74	0.077
**Weekly stair flights climbed, males only (n = 97)**	26	41.41	<10 - >200 flights	22.10–60.72	71	38.47	<10–150 flights	31.06–45.89	0.726
**Weekly stair flights climbed, females only (n = 148)**	29	61.51	<10 - >200 flights	38.85–84.17	119	35.61	<10 - >200 flights	29.68–41.54	0.031

^a^Independent samples t-test.

Statistical Significance (*P* ≤ 0.05).

Between group means for LBP were also analyzed separately for male and female respondents (Table 2). Three respondents declined to specify their gender and were therefore removed from this gender subgroup analysis. There was no significant difference between the LBP and NLBP groups for male respondents; however, a statistically significant difference in mean number of flights of stairs climbed per week was found among female respondents. Male NLBP respondents reported a mean of 41.41 (± 47.80; 95% CI, 22.10 to 60.72) flights climbed per week; and male LBP respondents reported 38.47 (± 31.32; 95% CI, 31.06 to 45.89) flights climbed per week, (*t*(95) = 0.352; *P* = 0.726). Female NLBP respondents reported a mean of 61.51 (± 59.57; 95% CI, 38.85 to 84.17) flights climbed per week; and female LBP respondents reported 35.61 (± 32.68; 95% CI, 29.68 to 41.54) flights climbed per week, (*t*(146) = 2.260; *P* = 0.031).

A one-way ANOVA was performed to compare mean flights of stairs climbed based on chronicity of LBP, which showed that differences in stairs climbed between these LBP chronicity groups was not significant, F(3,244) = 1.906, *P* = 0.129. When male and female respondents were analyzed separately, however, the one-way ANOVA was found to be significant for female respondent LBP chronicity, with F(3,144) = 3.592, *P* = 0.015. A Bonferroni post-hoc test was then performed and confirmed that the difference in stair flights climbed between NLBP and acute LBP respondents was statistically significant (*P* = 0.009), but no other between group differences were significant (Table 3).

**Table 3:** Between group differences in weekly stairs climbed with stratification for chronicity.

Variable	No LBP		Acute LBP		Sub-Acute LBP		Chronic LBP		*P*-value
	n	Mean	n	Mean	n	Mean	n	Mean	
**Weekly stair flights climbed, males & females (n = 248)**	56	51.62	138	36.48	26	41.18	28	41.31	0.129^a^
**Weekly stair flights climbed, males only (n = 97)**	26	41.41	54	39.63	8	30.22	9	38.89	0.900^a^
**Weekly stair flights climbed, females only (n = 148)**	29	61.51	83	33.92	17	36.22	19	42.46	0.015^a^
**Weekly stair flights climbed, females only No LBP vs. ACUTE (n = 112)**	29	61.51	83	33.92	n/a	n/a	n/a	n/a	0.009^b^

^a^One-way ANOVA

^b^Bonferroni post-hoc test.

Statistical Significance (*P* ≤ 0.05).

### Between group differences for other physical activities, psychosocial variables, and gender

The relationship between other types of physical activity, both recreational and occupational, and LBP prevalence, was also analyzed (Tables 4 and 5). The only significant between-group difference other than stair climbing was found for weekly resistance training among males and females combined, and for males only. This was measured on a 5 point Likert scale, with 1 being the lowest intensity and 5 being the highest intensity. For both genders combined, self-reported resistance training intensity was 2.98 (±1.37; 95% CI, 2.62 to 3.35) for NLBP respondents; and 2.53 (±1.31; 95% CI, 2.34 to 2.72) for LBP respondents, (*t*(244) = 2.206; *P* = 0.028). For males only, resistance training intensity was 3.12 (±1.37; 95% CI, 2.56 to 3.67) for NLBP respondents; and 2.47 (±1.38; 95% CI, 2.14 to 2.80) for LBP respondents, (*t*(94) = 2.037; *P* = 0.044).

**Table 4:** Between group differences in recall of distance and time spent on weekly exercise and physical activities.

Variable	No Low Back Pain				Low Back Pain				*P*-value^a^
	n	Mean	Range	95% CI	n	Mean	Range	95% CI	
**Weekly miles walked, males & females (n = 247)**	56	18.64	<1–42	15.74–21.54	191	17.01	1 - >50	15.52–18.49	0.307
**Weekly miles jogged/run, males & females (n = 248)**	56	5.98	<1 - >50	3.35–8.61	192	4.40	<1–30	3.53–5.27	0.258
**Weekly hours spent standing, males & females (n = 247)**	56	30.36	<1 - >50	26.01–34.70	191	32.01	1 - >50	29.92–34.10	0.470
**Weekly hours spent sitting, males & females (n = 247)**	56	36.09	<10 - >100	29.70–42.48	191	35.49	<10 - >100	32.93–38.05	0.862
**Weekly hours spent on physically demanding patient care, males & females (n = 248)**	56	2.88	0 - >40	1.15–4.61	192	3.57	0–25	2.87–4.27	0.394

^a^Independent samples t-test.

Statistical Significance (*P* ≤ 0.05).

**Table 5:** Between group differences in ranking of weekly exercise and physical activity intensity.

Variable	No Low Back Pain				Low Back Pain				*P*-value^a^
	n	Mean	Median	Mode	n	Mean	Median	Mode	
**Weekly aerobic exercise intensity, males & females (n = 247)** ^**b**^	56	3.45	3.00	3.00	191	3.18	3.00	3.00	0.188
**Weekly resistance exercise intensity, males & females (n = 246)** ^**b**^	56	2.98	3.00	4.00	190	2.53	2.00	2.00	0.028
**Weekly resistance exercise intensity, males only (n = 96)** ^**b**^	26	3.12	3.00	3 & 4.00^c^	70	2.47	2.00	1.00	0.044
**Weekly resistance exercise intensity, females only (n = 147)** ^**b**^	29	2.93	3.00	2 & 4.00^c^	118	2.55	2.00	2.00	0.165
**Weekly work-related physical activity intensity, males & females (n = 248)** ^**b**^	56	4.27	5.00	5.00	192	4.40	5.00	5.00	0.447
**Weekly housework and caregiving-related physical activity intensity, males & females (n = 247)** ^**b**^	55	3.02	3.00	2.00	192	3.17	4.00	4.00	0.430

^a^Independent samples t-test

^b^Ranked from 1 to 5, least intense to most intense

^c^Bi-modal frequency exists.

Statistical Significance (*P* ≤ 0.05).

Questions related to mental health and sleep factors were included in the survey, since these are known to be associated with LBP, and interactions as covariates were possible [46–48]. No significant differences between LBP and NLBP respondents were found for levels of depression, anxiety, or sleep quality for males and females combined. There was, however, a significant difference found between female LBP and NLBP respondents for sleep quality. An ANCOVA was run to determine the possible effect of age as a covariate in this analysis of sleep quality among female respondents, and the significant between-group difference was upheld. Details of these test results are contained in Table 6.

**Table 6:** Between group differences in depression, anxiety and sleep quality.

Variable	No Low Back Pain				Low Back Pain				*P*-value^a^
	n	Mean	Median	Mode	n	Mean	Median	Mode	
**Percentage of time was depressed during past year, males & females (n = 248)** ^**b**^	56	1.75	1.00	1.00	192	1.83	1.00	1.00	0.694
**Percentage of time was anxious during past year, males & females (n = 248)** ^**b**^	55	2.15	2.00	1.00	191	2.47	2.00	1.00	0.188
**Sleep quality during past year, males & females (n = 247)** ^**c**^	56	6.88	7.00	7.00	191	6.42	7.00	7.00	0.083
**Sleep quality during past year, females only (n = 147)** ^**c**^	29	7.38	8.00	7 & 8.00^d^	118	6.31	7.00	7.00	0.004

^a^Independent samples t-test

^b^Ranked from 1 to 7, from <10% to >50% of the time

^c^Ranked on 1 to 10 scale, from lowest to highest quality

^d^Bi-modal frequency exists.

Statistical Significance (*P* ≤ 0.05).

Because the association of stair flights climbed and LBP varied when male and female responses were analyzed separately, a male to female comparison of means was performed for all activities, with results found in Table 7. No significant differences were, however, found between males and females in time spent on, distance, or intensity of any activity.

**Table 7:** Between group activity differences for males versus females.

Variable	Males				Females				*P*-value^a^
	n	Mean	Range	95% CI	n	Mean	Range	95% CI	
**Weekly flights of stairs climbed (n = 245)**	97	39.26	<10 - >200 flights	31.96–46.56	148	40.69	<10 - >200 flights	34.11–47.26	0.779
**Weekly miles walked (n = 244)**	96	17.77	1 - >50	15.57–19.96	148	17.04	<1 - >50	15.40–18.69	0.596
**Weekly miles jogged/run(n = 245)**	97	4.66	<1–32	3.45–5.87	148	4.81	<1 - >50	3.52–6.09	0.875
**Weekly hours spent standing (n = 244)**	97	32.68	3 - >50	29.57–35.79	147	30.76	<1 - >50	28.36–33.15	0.329
**Weekly hours spent sitting(n = 244)**	97	36.20	<10 - >100	32.11–40.28	147	35.37	<10 - >100	32.26–38.47	0.746
**Weekly hours spent on physically demanding patient care (n = 245)**	97	2.89	0–20	2.09–3.68	148	3.66	0 - >40	2.70–4.62	0.259
**Weekly aerobic exercise intensity (n = 244)** ^**b**^	96	3.30	n/a	n/a	148	3.22	n/a	n/a	0.648
**Weekly resistance exercise intensity (n = 243)** ^**b**^	96	2.64	n/a	n/a	147	2.62	n/a	n/a	0.910
**Weekly work-related physical activity intensity (n = 245)** ^**b**^	97	4.41	n/a	n/a	148	4.33	n/a	n/a	0.559
**Weekly housework and caregiving-related physical activity intensity (n = 244)** ^**b**^	97	2.97	n/a	n/a	147	3.22	n/a	n/a	0.121

^a^Independent samples t-test

^b^Ranked from 1 to 5, least intense to most intense.

Statistical Significance (*P* ≤ 0.05).

## Discussion

In the present sample of US physical therapists, a statistically significant association was found showing fewer weekly stair flights climbed among female respondents with LBP. The one-year prevalence of LBP was lower among female but not male respondents who climbed more stairs, and a further and stronger association was found for the condition of acute LBP and stair climbing in female respondents, but not for sub-acute or chronic LBP (Tables 2 and 3).

The potential cardiovascular health benefits of stair climbing are now widely accepted but, to our knowledge, this is the first study to examine the impact of stair climbing on LBP morbidity. The results of the London Transport Worker’s study [2,49] showed significantly less cardiovascular mortality and morbidity among bus conductors compared to drivers; with even greater mean differences when double-decker bus conductors were compared to single story bus conductors. Since that time, these results have been confirmed showing an inverse relationship between stairs climbed and all-cause but not cardiovascular mortality in analyses based on the Harvard Alumni Health Study [3], and the UK Biobank Study [4]. Other studies have shown improved endothelial function in individuals with hypertension [5], and decreased peripheral vascular disease mortality [50], with an increase in stairs climbed.

This study of the potential impact of stair climbing on LBP builds on recent research showing an association between LBP and hip extensor CSA and weakness [18,20,51]. These studies were in turn inspired by extensive prior research showing decreased CSA in the multifidus and other trunk muscles of people with LBP [52–56]. Regional interdependency among the joints of the lower limb and the lumbar spine is now a major area of inquiry, with studies finding weakness and EMG changes in the hip musculature of individuals with both knee pain and LBP [57–60].

Association but not causation can be inferred by the present type of cross-sectional study. Although significant between-group differences were seen with subsets of our sample, we cannot determine if stair climbing acted as a kind of protective factor for our female respondents with regard to LBP; or whether, for example, this is a case of reverse causation whereby females with LBP in our sample chose to climb fewer stairs (e.g., take the elevator more), to avoid pain caused by this activity, or due to fear-avoidance behavior. Additionally, an unmeasured covariate may be affecting the LBP prevalence rate among part of our sample.

Even if one were to assume that stair climbing has a protective effect on LBP, it is puzzling that women would show a significantly stronger LBP response to this activity than men. Hypotheses for potential differences between men and women may be related to biomechanical differences in the way women climb stairs: for example, sex differences may exist in moments of trunk and hip flexion and extension during stair climbing. Known sexual dimorphism in lumbar lordosis angle [61,62] may be a factor if, for example, stair climbing accentuates lumbar extension. The fact that women continue to perform a greater percentage of household chores than male partners in most western cultures [63,64], may have contributed to a gender-based difference in response to stair climbing. A task such as doing laundry, which can involve carrying heavy baskets of clothes up and down stairs, would make this activity more physically demanding, activating GM and paraspinal muscles to a greater degree. Information regarding items carried during the performance of stair climbing was not gathered as a part of the present survey, but should be included in future study designs.

Similarly, there is no easy explanation for the stronger association between stairs climbed and acute versus subacute and chronic LBP in our female respondents. One interpretation would be that stair climbing would seem to have a preventative, but not a treatment, effect once LBP has reached the subacute phase in our female respondents. There are established gender differences in the experience of pain, but accurately measuring these differences is confounded by the inability of many studies to differentiate between true pain incidence and the willingness to report pain [65]. Manifestation of fear-avoidance behaviors, including hypervigilance in avoiding potential pain triggers, may vary by gender, but research to date is inconclusive [66]. A eustress response to LBP, which would entail greater resilience, a more positive attitude, and higher overall activity levels in the face of pain [67,68], may partially explain the higher number of stairs climbed with longer duration of symptoms among our female respondents. Buchman et al [69] identified an important eustress versus distress response to chronic LBP in some study participants, with eustress leading to better rehabilitation outcomes; however, they did not report on gender differences in their study results. Understanding the interaction of these types of pain responses with our cohort’s reported pain levels was beyond the scope of the current research and would be best assessed through a longitudinal study design.

When other activities were analyzed, the only other significant between-group difference was found for male respondent LBP and weekly resistance exercises performed, with a lower prevalence of LBP associated with more intense resistance exercise. The mechanism of a possible greater protective effect of resistance training in men versus women is also unclear. Both upper and lower extremity resistance exercises are considered a form of lumbar stabilization exercise [70], and lumbar stabilization exercises have been shown to be an effective intervention for certain types of LBP [14,71,72]. Yet this effect should also, therefore, have been evident in female respondents.

One unusual finding from our research is that hours spent sitting per week was not correlated with LBP prevalence in our sample. Sitting is a known risk factor for certain types of LBP [73–75], in particular discogenic or posterior derangement back pain types [15,76]. The fact that our sample consisted entirely of physical therapists, most of whom work in an adult orthopedic outpatient setting, may explain this anomalous finding. Orthopedic physical therapists with LBP would be expected to try and eliminate lifestyle risk factors such as prolonged sitting, from their own daily activities, as this is part of regular patient education for LBP patients [77,78].

Any understanding of a possible causal mechanism connecting stair climbing and LBP is in the rudimentary stages. Prior research conducted by our team [21] explored possible etiologies supporting a relationship between GM strength and LBP, employing a strategy based on analysis of the anatomical and biomechanical relationships that exist in the gluteal and lumbar region. Multiple causal mechanisms were hypothesized including: strong GM muscles may compensate for weaker paraspinal muscles during lifting tasks; stair climbing and other GM exercises may elicit lumbar stabilization and/or increase the lumbar lordosis angle; GM attachments to the thoracolumbar fascia (TLF) could facilitate TLF axial compression and power of erector spinae muscles; and GM contraction may stimulate TLF sensory receptors that facilitate spinal stabilization and motor control. All of the above are mere hypotheses and suggest possible directions for future research into the connection between LBP, GM, and stair climbing.

### Limitations

The one-year prevalence period used in the present study will inevitably have had some impact on the reliability of our data, due to recall bias [79,80]. Self-report of both medical history and activity levels is also, for any prevalence period, less reliable than quantitative measurement of these variables, and will have impacted the accuracy of the data we obtained from our survey respondents [81,82]. A larger sample size, especially for our NLBP control group, would possibly have unmasked further between-group differences. Our assumption that physical therapists have a similar socioeconomic status may not hold true when comparing regional salary and cost of living differences, or overall household income variations. Financial status can affect LBP prevalence [25], and our survey design did not capture financial information, so this may be an unmeasured covariate. Our results can also not be considered generalizable to non-physical therapists. Finally, the association between stair climbing and LBP in the female respondents in the present sample cannot be interpreted as causal, due to our cross-sectional study design.

## Conclusions

A greater number of weekly stairs climbed was associated with a lower LBP prevalence among female physical therapists, with a stronger association seen when comparing acute LBP to no LBP. Prospective, randomized, longitudinal research assessing the impact of stair climbing on LBP is required to rule out the impact of unmeasured covariates, bias in self-reported activity and health variables, and the impact of possible reverse causation on these outcomes.

## Supporting information

S1 Checklist STROBE statement—checklist of items that should be included in reports of observational studies. (PDF)

S1 Appendix Low back pain & stair climbing survey questions. (DOCX)

S1 File (XLSX)
