# Supplementary material for: Greater number of weekly stairs climbed is associated with lower low back pain prevalence among female but not male physical therapists
Source: PLoS One. 2023 Oct 5;18(10):e0292489. doi: 10.1371/journal.pone.0292489 (PMC10553291; doi:10.1371/journal.pone.0292489)
Supplement: S1 Appendix — (DOCX) [file pone.0292489.s002.docx]

**Appendix: Low Back Pain and Stair Climbing Survey Questions**

| **Question Text** | ***Answer Format*** |
| --- | --- |
| - Q1 I am a Physical Therapist and I consent to participate in this survey. | *Yes/No* |
| - Q2 In what year were you born? | *Drop Down Boxes* |
| - Q3 What is your gender? | *Drop Down Boxes* |
| - Q4 What is your Height in INCHES? | *Drop Down Boxes* |
| - Q5 What is your weight, in pounds? | *Drop Down Boxes* |
| - Q6 In general, I would say that my health is: | *Drop Down Boxes* |
| - Q7 In the Past 12 Months, have you experienced any type of Pain ranking >5/10 on the numeric scale that LASTED > 2 weeks? | *Yes/No* |
| - Q7a Please provide General Location of Pain (eg, knee, headache). | *Text Entry* |
| - Q8 How many Face-to-Face Medical (with eg and MD, CNP, or PA)Visits have you had in the past 12 months? | *Drop Down Boxes* |
| - Q9 How many Telehealth Medical Visits (with eg and MD, CNP, or PA) have you had in the past 12 months? | *Drop Down Boxes* |
| - Q10 In the Past 12 Months, have you undergone ANY Ambulatory or Inpatient Surgery? (Don’t include screening procedures like mammograms or colonoscopies) | *Yes/No* |
| - Q11 Have you EVER had any of the following conditions? Check All that apply. | *Check Boxes* |
| - Q12 During the Past 12 Months, have you experienced ANY Low Back Pain (LBP)? LBP is defined as pain located between the 12th rib and the gluteal fold that may or may not radiate into the proximal or distal lower extremity. | *Yes/No* |
| - Q13 What was the Duration of your LBP in WEEKS? Check One. | *Drop Down Boxes* |
| - Q14 Using the 0 to 10 numeric pain scale, How Painful was your LBP on your worst day, WITHOUT pain medication? | *0 – 10 Scale* |
| - Q15 Did your LBP in the Past 12 Months cause you to Limit your Physical Activities in any way? | *Drop Down Boxes* |
| - Q16 Number of LIFETIME Prior Episodes of LBP lasting > one week. | *Drop Down Boxes* |
| - Q17 Did you receive or self-administer Any Treatment for your LBP in the Past 12 Months? | *Yes/No* |
| - Q18 What type of NON- PHYSICAL THERAPY treatment did you have for LBP In the Past 12 Months? Check all that apply. | *Check Boxes* |
| - Q19 What type of PHYSICAL THERAPY treatment did you have for LBP In the Past 12 Months? ? Can be self-administered. Check all that apply. | *Check Boxes* |
| - Q20 In what Type of Home do you live? | *Drop Down Boxes* |
| - Q21 On What floor do you live? (Specify number of Floors) | *Drop Down Boxes* |
| - Q22 Do you have a working elevator in your apartment building? | *Yes/No* |
| - Q23 During the Past 12 Months, what was your Average level of RECREATIONAL Aerobic physical activity based on the options below. Please choose one answer, and provide your BEST ESTIMATE Without using a Fitbit or other Activity Monitor. | *Check Boxes* |
| - Q24 During the Past 12 Months, what was your Average level of RECREATIONAL Strength Training or Resistance Exercise activity based on the options below. Please choose one answer, and provide your BEST ESTIMATE Without using a Fitbit or other Activity Monitor. | *Check Boxes* |
| - Q25 During the Past 12 Months, what was your Average level of WORK-RELATED PHYSICAL ACTIVITY as a PT? Please choose one answer, and provide your BEST ESTIMATE Without using a Fitbit or other Activity Monitor. | *Check Boxes* |
| - Q26 During the Past 12 Months, what was your Average level of PHYSICAL ACTIVITY related to CAREGIVING (eg for an adult or child family member) and/or HOUSEWORK (eg, cooking and cleaning) IN YOUR HOME based on the options below. Please choose one answer, and provide your BEST ESTIMATE Without using a Fitbit or other Activity Monitor. | *Check Boxes* |
| - Q27 During the Past 12 Months, how many FLIGHTS OF STAIRS did you climb UP in the Average Week? Please provide your BEST ESTIMATE Without using a Fitbit or other Activity Monitor. - One flight of stairs is 10-13 steps, with grossly standard 8 inch rise and 9 inch run. Please include stairs at home, work, during your commute and for recreation/exercise. For example, if you live in a 3rd floor apartment and went out, on average, only one time per day, your answer would be 3 flights per day, so 21 flights climbed per WEEK. | *Drop Down Boxes* |
| - Q28 During the Past 12 Months, how many Miles did you WALK in an Average Week? Please include walking at home, work, during your commute and for recreation/exercise? Please provide your BEST ESTIMATE Without using a Fitbit or other Activity Monitor. | *Drop Down Boxes* |
| - Q29 During the Past 12 Months, how many Miles did you JOG or RUN in an Average Week? Please provide your BEST ESTIMATE Without using a Fitbit or other Activity Monitor. | *Drop Down Boxes* |
| - Q30 During the Past 12 Months, how many HOURS in an Average Week did you spend STANDING (not including when you were walking or running)? Please include standing at home, work, during your commute, and for recreation or exercise. Please provide your BEST ESTIMATE Without using a Fitbit or other Activity Monitor. | *Drop Down Boxes* |
| - Q31 During the Past 12 Months, how many HOURS in an Average Week did you spend SITTING? Please include sitting at home, work, during your commute, and for recreation. Please provide your BEST ESTIMATE Without using a Fitbit or other Activity Monitor. | *Drop Down Boxes* |
| - Q32 During the Past 12 Months, how many HOURS in an Average Week did you spend performing Transfers, Bed Mobility, or other Physically Demanding Patient Handling in the course of your work as a PT)? Please provide your BEST ESTIMATE Without using a Fitbit or other Activity Monitor. | *Drop Down Boxes* |
| - Q33 During the Past 12 Months, for what PERCENTAGE (%) of the time did you feel DEPRESSED? Please provide your BEST ESTIMATE. | *Drop Down Boxes* |
| - Q34 During the Past 12 Months, for what PERCENTAGE (%) of the time did you feel ANXIOUS? Please provide your BEST ESTIMATE. | *Drop Down Boxes* |
| - Q35 During the Past 12 Months, how would you rank your Overall SLEEP QUALITY on Average? Please provide your BEST ESTIMATE. Please drag the cursor below to show your sleep quality. | *0 – 10 Scale* |
| - Q36 During the Past 12 Months did you work as a physical therapist? | *Drop Down Boxes* |
| - Q37 How many years have you been a physical therapist? | *Drop Down Boxes* |
| - Q38 During the Past 12 Months in What Physical Therapy work Setting and for HOW MANY HOURS did you practice in an Average Week? Check all settings and provide weekly hours for all that apply. | *Check Boxes and Text Entry* |
| - Q39 Did you move into a new home in the last 12 months? | *Yes/No* |
| - Q40 In which state is your primary residence during the majority of the past 12 months? | *Drop Down Boxes* |
| - Q41 What is the zip code of your primary residence during the majority of the past 12 months? | *Text Entry* |
